# Positive Selection of ORF1ab, ORF3a, and ORF8 Genes Drives the Early Evolutionary Trends of SARS-CoV-2 During the 2020 COVID-19 Pandemic

**DOI:** 10.3389/fmicb.2020.550674

**Published:** 2020-10-23

**Authors:** Lauro Velazquez-Salinas, Selene Zarate, Samantha Eberl, Douglas P. Gladue, Isabel Novella, Manuel V. Borca

**Affiliations:** ^1^Foreign Animal Disease Research Unit, USDA/ARS Plum Island Animal Disease Center, Greenport, NY, United States; ^2^College of Veterinary Medicine, Kansas State University, Manhattan, KS, United States; ^3^Posgrado en Ciencias Genómicas, Universidad Autónoma de la Ciudad de Mexico, Mexico City, Mexico; ^4^Department of Psychological Science, Central Connecticut State University, New Britain, CT, United States; ^5^Independent researcher, Toledo, OH, United States

**Keywords:** evolution, epistasis, positive selection, COVID-19, SARS-CoV2

## Abstract

In this study, we analyzed full-length SARS-CoV-2 genomes from multiple countries to determine early trends in the evolutionary dynamics of the novel COVID-19 pandemic. Results indicated SARS-CoV-2 evolved early into at least three phylogenetic groups, characterized by positive selection at specific residues of the accessory proteins ORF3a and ORF8. Also, we are reporting potential relevant sites under positive selection at specific sites of non-structural proteins nsp6 and helicase. Our analysis of co-evolution showed evidence of epistatic interactions among sites in the genome that may be important in the generation of variants adapted to humans. These observations might impact not only public health but also suggest that more studies are needed to understand the genetic mechanisms that may affect the development of therapeutic and preventive tools, like antivirals and vaccines. Collectively, our results highlight the identification of ongoing selection even in a scenario of conserved sequences collected over the first 3 months of this pandemic.

## Introduction

The first case of pneumonia confirmed to be caused by the novel virus severe acute respiratory syndrome coronavirus 2 (SARS-CoV-2) was a patient admitted in a hospital of Wuhan, Hubei province, China on December 12, 2019 (Wu et al., 2020). As of April 9, 2020, the World Health Organization (WHO) has confirmed 1,439,516 cases, 85,711 deaths, and the presence of COVID-19 in 209 countries, areas, or territories. Of the confirmed cases, 71% are from seven countries: United States of America (395,030), Spain (146, 690), Italy (139, 422), Germany (108,202), China (83,249), France (81,095), and Iran (66,220). As of the writing of this report, the number of COVID-19 cases continue to increase worldwide, with multiple epicenters. Remarkably, by the time of the revision of this manuscript (September 21, 2020), the number of both confirmed cases and deaths has dramatically increased to 30,905,162 and 958,703, respectively, becoming the Americas the center of the pandemic with 15,580,622, and 530,373 confirmed cases and deaths, respectively, and corroborating the huge impact of this pandemic for the public health around the world.^1^

The International Committee on Taxonomy of Viruses (ICTV) initially named this pathogen 2019-nCoV (also referred to as COVID-19 by WHO) and included it within the *Coronaviridae* viral family (Coronaviridae Study Group of the International Committee on Taxonomy of Viruses, 2020). Later, based on the close phylogenetic relationship of COVID-19 with other human and bat SARS-CoVs, ICTV renamed the virus as SARS-CoV-2 (Coronaviridae Study Group of the International Committee on Taxonomy of Viruses, 2020).

The *Coronaviridae* family encompasses a group of single-stranded, positive-sense RNA viruses with a genome length varying between 27 and 32 kb. These are zoonotic viruses with the potential to infect humans and animals. Coronaviruses may cause acute and chronic respiratory, enteric, and central nervous system infections (Weiss and Navas-Martin, 2005; Phan et al., 2018). In the case of SARS-CoV-2, a meta-analysis of 50,466 patients indicates that fever and cough are the most common symptoms (95% CI: 81.8–94.5% and 65.7–78.2%, respectively; Sun et al., 2020). The disease may worsen, and the percentages of severe cases and fatality rate vary between 12.7 and 24.3% and 2.7 and 6.1% (95% CI), respectively (Sun et al., 2020). Interestingly, new clinical evidence obtained by the time of this revision shows the ability of SARS-CoV-2 to produce arrhythmia, septic shock, coagulation dysfunction, and multiple organ functional failure (Wang et al., 2020).

The genome organization of SARS-CoV-2 is similar to viruses from the genus *Betacoronavirus*, one of the four genera included in the *Coronaviridae* subfamily Orthocoronavirinae. The ~29,903 nucleotide (nt) genome is organized as follows, 5' to 3': replicase ORF1ab, S (encoding the structural spike glycoprotein), ORF3a (ORF3a protein), E (structural envelope protein), M (structural membrane glycoprotein), ORF6 (ORF6 protein), ORF7a (ORF7a protein), ORF7b (ORF7b protein), ORF8 (ORF8 protein), N (structural nucleocapsid phosphoprotein), and ORF10 (ORF10 protein). ORF1ab (~21,291 nt) encodes 16 non-structural proteins: leader protein, nsp2, nsp3, nsp4, 3C-like proteinase, nsp6, nsp7, nsp8, nsp9, nsp10, RNA-dependent RNA polymerase, helicase, 3' to 5' exonuclease, endoRNAse, 2'-O-ribose methyltransferase, and nsp11 (Wu et al., 2020).

Much speculation regarding the origin of SARS-CoV-2 emanates from unfounded theories, such as a man-made laboratory origin; however, a recent study supports the hypothesis that SARS-CoV-2 was the result of cross-species transmission followed by natural selection in the novel human host (Andersen et al., 2020). This hypothesis is strongly supported by studies examining amino acid differences between SARS-CoV-2 and some phylogenetically related betacoronaviruses (e.g., Bat-RatG13 isolate and the human SARS-CoV isolate Urbani) at the receptor-binding domain (RBD) of the spike protein, where such differences seem to increase the ability of SARS-CoV-2 to bind to the human receptor angiotensin-converting enzyme 2 (ACE2; Andersen et al., 2020). This increased affinity for binding ACE2 might help to explain the infectiousness of SARS-CoV-2 in human populations (Wan et al., 2020).

Considering the extraordinary plasticity shown by other human viral RNA pathogens, for example, HIV-1, Influenza viruses, SARS-CoV, and hepatitis C virus, to undergo adaptative changes to evade innate and adaptive immune responses, develop drug resistance, or establish an infection in a new host (Frost et al., 2018), multiple questions arise regarding the adaptative changes that SARS-CoV-2 has undergone during the pandemic. SARS-CoV-2 has spread throughout many countries resulting in the infection of people with diverse immunological backgrounds and demographics (age, sex, environmental conditions, etc.) that potentially impose significant selective pressures on SARS-CoV-2.

Here, we evaluate the phylogenetic and evolutionary dynamics of SARS-CoV-2 during the early phase of the COVID-19 pandemic. Using different analyses based on a codon-based phylogenetic framework, we identified critical sites in the genome undergoing positive selection, which might favor viral divergence and emergence of multiple viral variants. Our findings are discussed in terms of the potential effects that the early evolution of SARS-CoV-2 might have on the outcome of this pandemic.

## Materials and Methods

### Data Collection

**Figure 1:**
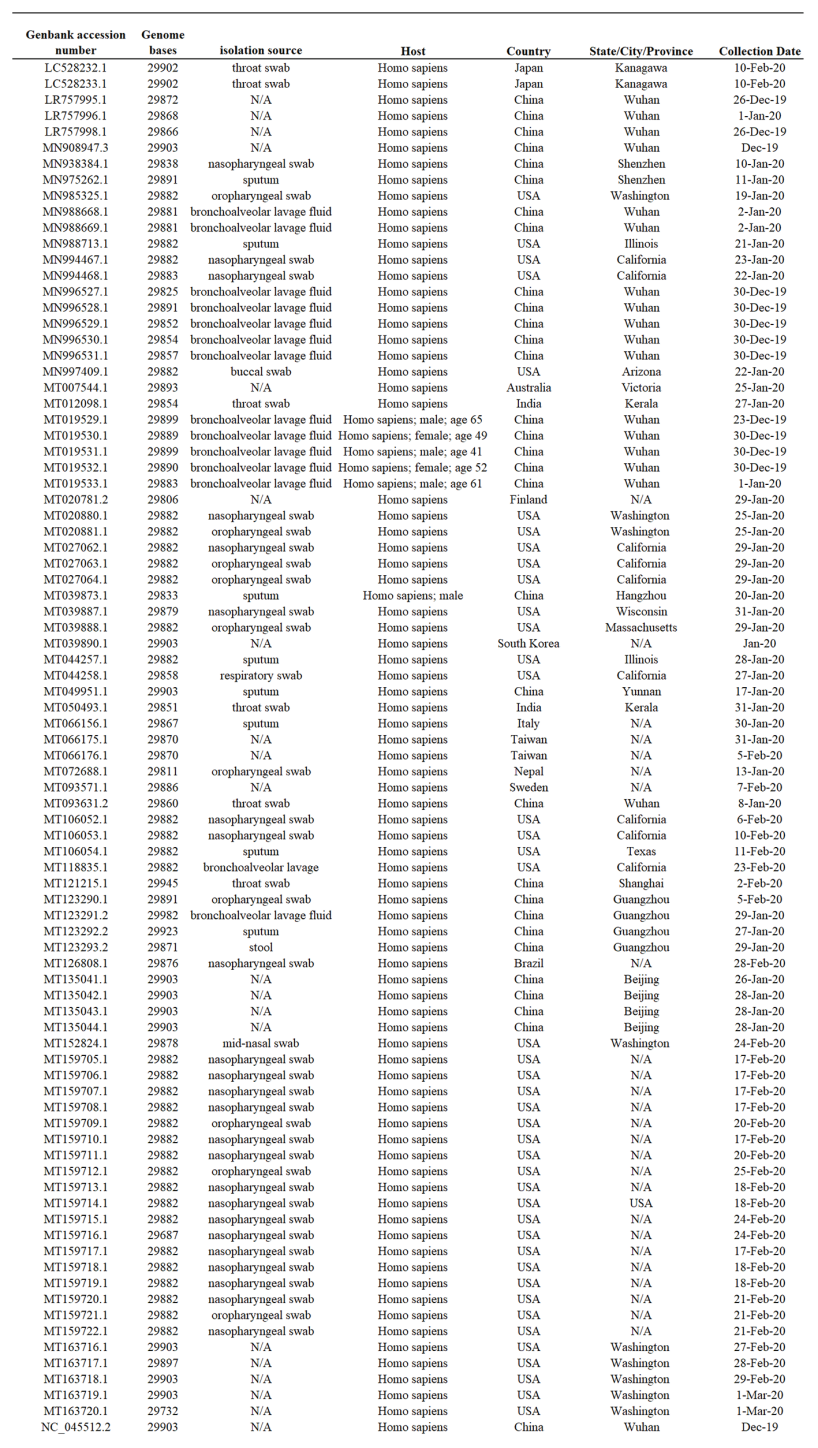
Sample summary. Description of the 86 SARS-Cov-2 full-length genome sequences included in this study. All sequences were obtained form I from the NCBI severe acute respiratory syndrome coronavirus 2 (SARS-Cov-2) data hub, accession number, genome length, isolate name, source, host, and country of origin are provided. N/A indicates information not available.

Eighty-six full-length SARS-CoV-2 genomes representing early viral isolates from patients living in diverse geographic regions were used for this study. Viral sequences available to be downloaded from the NCBI SARS-CoV-2 data hub as of March 10, 2020 represent the total number of full-length viral genomes at the time that the analysis was conducted (Figure 1).

### Phylogenetic Analysis

A phylogenetic tree of SARS-CoV-2 was reconstructed using a Bayesian approach on the program MrBayes 3.2.7 (Huelsenbeck and Ronquist, 2001; Ronquist and Huelsenbeck, 2003). For this propose, Hasegawa-Kishino-Yano 85 (HKY85) was used as nucleotide substitution model (Hasegawa et al., 1985). This model was chosen based on the Bayesian information criterion (BIC) score (84484.082), and in its availability on MrBayes. HKY85 represented the third best option among 24 substitution models (Supplementary Figure 1). This analysis was conducted on the software Mega 7 (Kumar et al., 2016)

Settings on MrBayes included: number of substitutions types (Nst) = 2 (allowing transitions and transversions have potentially different rates), rates = Gamma, and Markov Chain Monte Carlo (MCMC) = 100,000,000. The run was diagnosed using Tracer 1.7.1 (Rambaut et al., 2018) to ensure an ESS larger than 200. The tree was built using a 15% burnin proportion and the half compatible rule in order to collapse all the nodes with a posterior probability lower than 0.5. The final tree was visualized using Figtree 1.4.3.^2^

### Rates of Evolutionary Change

Rates of evolutionary change of SARS-CoV-2, expressed as substitutions/site/year, were calculated using the programs BEAST2.4.3, BEAUti, and Tracerr, introducing the sampling date as a trait (Drummond et al., 2012). MCMC was run for 100 million generations, using the HKY85 substitution model and a gamma distribution with four categories as the site heterogeneity model. The resulting file was analyzed with Tracer 1.7 to check for convergence and to determine the evolutionary rate.

### Population Structure Analysis

The extent of genetic differentiation (population structure) between different phylogenetic groups of SARS-CoV-2 was evaluated by the fixation index (*F*_ST_; Hudson et al., 1992). This test was developed by Sewall Wright and determines the overall genetic divergence among subpopulations by evaluating the difference between mean pairwise intra-subpopulation diversity with mean pairwise inter-subpopulation diversity in order to establish population structure. *F*_ST_ values range between 0 and 1, reflecting undifferentiated to fully differentiated populations, respectively. Overall, a value <0.33 for viral populations suggests lack of genetic divergence between subpopulations (Wei et al., 2009; Zu et al., 2019). Analysis was conducted on the software HyPhy (Pond et al., 2005), and a randomization test with 1,000 replicas was carried out to determine statistical significance (*p* < 0.001).

### Pairwise Distance Calculations

Nucleotide and amino acid pairwise distance calculations among SARS-CoV-2 sequences were conducted using the SSE 1.3 Sequence Distances program (Simmonds, 2012), as previously described for the genome characterization of hepatitis C virus genotype 7 (Salmona et al., 2016). However, based on the high level of identity found in the set of SARS-CoV-2 sequences evaluated in our research, we decided to use a sliding window of 50 nt, instead of 300 nt as reported by Salmona et al. (2016), with a shift of 25 nt. Additionally, *p*-distances in nucleotide and amino acid sequences between phylogenetic groups were calculated using MEGA 7 (Kumar et al., 2016).

### Evolutionary Rate per Site Analysis

Mean (relative) evolutionary rates for each site in the alignment were estimated under the General Time Reversible model, including all three codon positions. These rates were scaled, considering the average evolutionary rate across all sites is 1. This means that sites showing a rate < 1 are evolving slower than average, and those with a rate > 1 are evolving faster than average. This analysis was conducted using MEGA 7 (Kumar et al., 2016).

### Inference of Selective Pressures

Since natural selection can be manifested as different modes (diversifying, directional, or purifying), we used a combination of different evolutionary analyses to enhance the detection of relevant sites in the genome of SARS-CoV-2 experiencing diversifying (positive) and purifying (negative) selection: single likelihood ancestor counting (SLAC; Kosakovsky Pond and Frost, 2005), fixed effects likelihood (FEL; Kosakovsky Pond and Frost, 2005), mixed effects model of evolution (MEME; Murrell et al., 2012), and fast unbiased Bayesian approximation (FUBAR; Murrell et al., 2013). These methods use a maximum likelihood or Bayesian approach (FUBAR) to infer nonsynonymous (dN) and synonymous (dS) substitution rates on a per site basis for given coding alignment and corresponding phylogeny (Weaver et al., 2018). SLAC, FEL, and FUBAR were methods used to identify sites experiencing pervasive diversifying or purifying selection, while MEME was used to detect sites experiencing both pervasive and episodic diversifying selection (Spielman et al., 2019).

The presence of recombination in the sequence dataset potentially affecting the detection of positive selection was assessed using the algorithm genetic algorithm for recombination detection (GARD; Kosakovsky Pond et al., 2006). All methods were performed on the adaptive evolution server Datamonkey 2.0 (Weaver et al., 2018).

Evidence of directional selection was assessed on amino acid sequences using the directional evolution of protein sequences (DEPS) method, implemented on the Datamonkey webserver (classic; Delport et al., 2010). This method is a model-based phylogenetic maximum likelihood test that looks for evidence of preferential substitution toward a given residue at individual positions of a protein alignment (Kosakovsky Pond et al., 2008). DEPS has the ability to overcome diverse evolutionary scenarios that confound most existing evolutionary tests (Kosakovsky Pond et al., 2008). For additional details about the evolutionary methods used in this research, see Supplementary Figure 2.

### Coevolution Analysis

Evidence of coevolution among different sites in the SARS-CoV-2 genome was evaluated using the method Bayesian Graphical Models for co-evolving sites (BGM; Poon et al., 2007). This method detects coevolutionary interactions between amino acids in a protein, where amino acid substitutions are mapped to branches in the phylogenetic tree.

### Blosum 62 Substitution Matrix

Blosum 62 substitution matrix (BSM62) was used to infer the nature of amino acid replacements found during the evolutionary analysis of SARS-CoV-2, where positive values reflect that the substitution is most likely a product of random substitution, while negative values may be indicative of selection (Henikoff and Henikoff, 1992).

## Results

### Phylogenetic Dynamics of SARS-CoV-2

**Figure 2:**
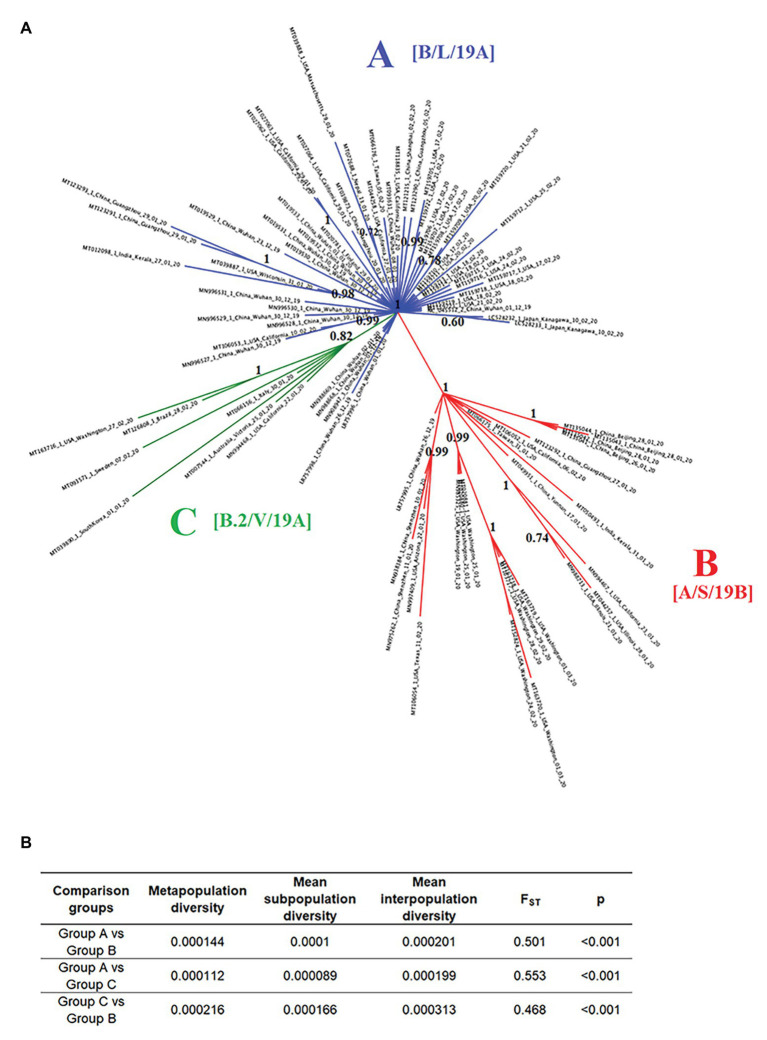
Phylogeny and population structure analysis of SARS-Cov-2. **(A)** Bayesian tree reconstructed using 86 SARS-Cov-2 full-length genomes collected from patients naturally infected at different countries, showing the existence of three phylogenetic groups: A (blue), B (red), and C (green). Numbers over the nodes represent their posterior probability. Information in the brackets corresponds with the current nomenclature proposed to describe different lineages reported in our study (https://www.gisaid.org/references/statements-clarifications/clade-and-lineage-nomenclature-aids-in-genomic-epidemiology-of-active-hcov-19-viruses/). **(B)** Intra- and inter-subpopulation diversity among phylogenetic groups was compared to determine the extent of population structure. *F*_ST_ values >0.33 (*p* < 0.001) were consider significant.

To evaluate potential divergence events of SARS-CoV-2, indicating the rise of new variants early during the pandemic, we reconstructed the evolution of SARS-CoV-2 using full-length genome sequences of viruses collected between late December of 2019 and early March of 2020 from patients infected in different countries around the world. The results of the phylogenetic analysis demonstrate the rapid divergence of SARS-CoV-2 into three distinct phylogenetic groups (A, B, and C). The divergence of these groups was strongly supported by high values of posterior probability that range from 0.82 to 1 (Figure 2A). Interestingly, *F*_ST_ analysis also supported the early divergence of these three groups, showing statistical significant values (*p* < 0.001), and *F*_ST_ values >0.33 between all group comparisons (~0.51 s 0.4; Figure 2B).

Group A includes one of the first viral sequences generated during the outbreak in Wuhan, China, collected on December of 2019 (NC_045512.2), as well as multiple viral isolates from different Chinese provinces. The position of these sequences among multiple branches within the Group A cluster suggests the emergence of multiple viral variants in China, especially from Wuhan before the start of the global pandemic. Furthermore, the basal branch position of some of these variants indicates that they were the ancestors of viral isolates obtained from patients in the United States, Japan, Finland, Taiwan, Nepal, and India between January and February of 2020.

Similarly, in the Group B cluster, we found viral isolates from multiple Chinese provinces between December of 2019 and January of 2020. These isolates are likely ancestors of viral isolates recovered from patients in the United States, India, and Taiwan between January and March of 2020. Interestingly, one isolate from Wuhan (LR757995.1) is part of the Group B cluster, supporting the hypothesis that multiple viral variants emerged in China before the start of the pandemic.

The Group C cluster was the only cluster that did not contain sequences from China. This cluster includes viral isolates collected from the United States, Italy, Australia, Sweden, Brazil, and South Korea between January and February of 2020. The absence of viral isolates from China and the increased genetic distance from Group A suggests that the emergence of these variants might have come from a second wave of transmission outside of China after the start of the pandemic.

Importantly, by the time of the revision of this manuscript (September 20, 2020), different classifications have been published regarding the clade and lineage nomenclature of SARS-CoV-2. In this sense, the groups arbitrarily named and reported in our study as A, B, and C are now classified as follows: A as B, L, or 19A, B as A, S, 19B, and group C as B.2, V, or 19A (Alm et al., 2020).

### Evolutionary Divergence in the Genome of SARS-CoV-2

**Figure 3:**
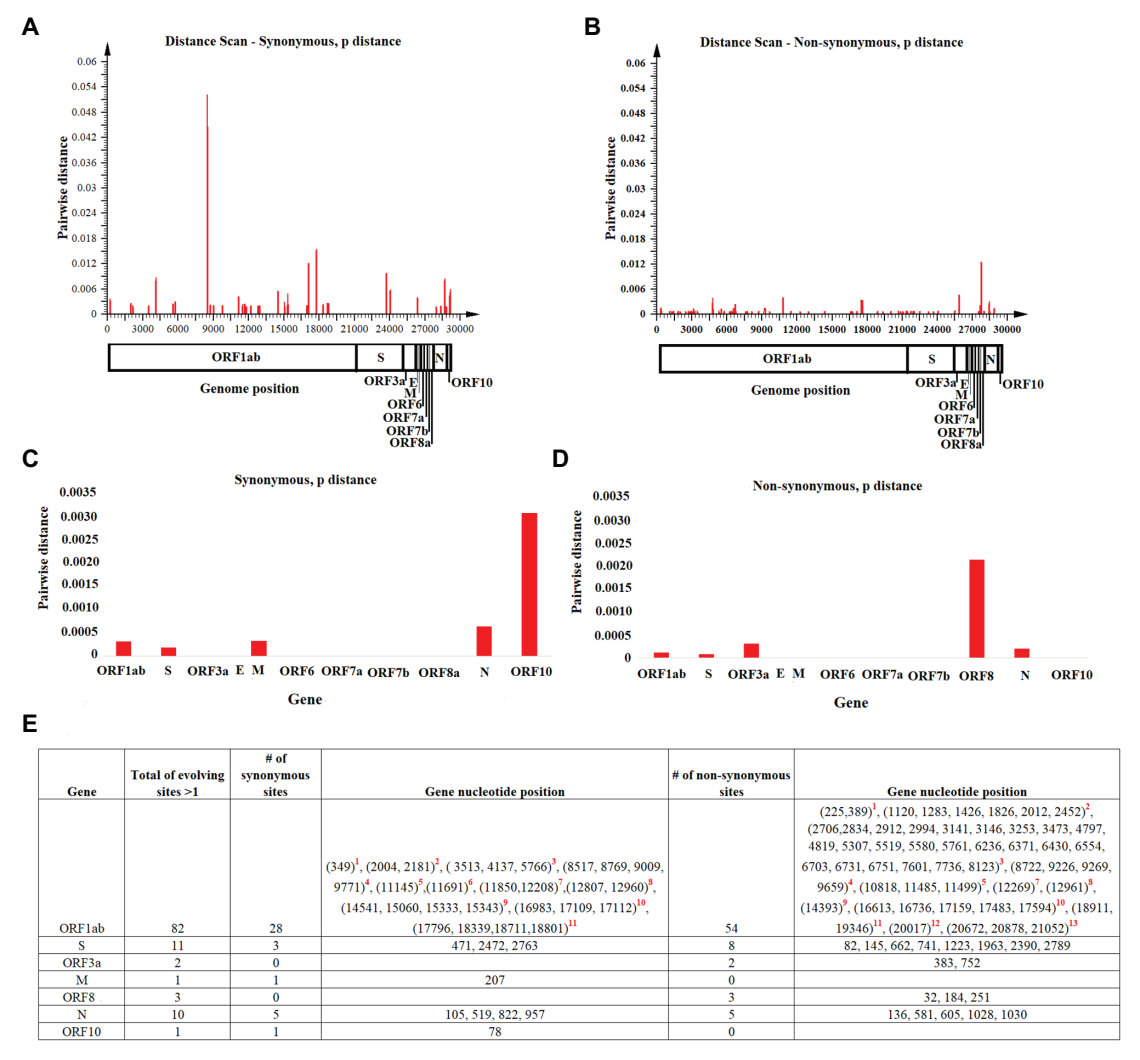
Pairwise distance analysis. Pairwise distance analysis at **(A)** synonymous and **(B)** non-synonymous nucleotide sites was conducted using the program Sequence Distances (software SSE). Red bars represent pairwise distance comparisons using a sliding window of 50 nucleotides. Average nucleotide pairwise distance for different genes is shown at **(C)** synonymous and **(D)** non-synonymous sites. **(E)** Fast-evolving synonymous and non-synonymous sites at each coding region are shown. For these sites, evolutionary rates oscillated between 4.97 and 4.95. Red numbers represent nucleotides at: (1) leader protein, (2) nsp2, (3) nsp3, (4) nsp4, (5) nsp6, (6) nsp7, (7) nsp8, (8) nsp10, (9) RNA independent polymerase, (10) helicase, (11) 3' to 5' exonuclease, (12) endoRNAse, and (13) 2'-O-ribose methyltransferase.

Once we reconstructed the phylodynamic of SARS-CoV-2 isolates obtained early during the pandemic event, we attempted to determine which nucleotide positions in the SARS-CoV-2 genome were related to the early divergence of this virus. Overall, the evolutionary rate of SARS-CoV-2 is 1.15 × 10^−3^ substitutions/site/year (95% HPD 7.41 × 10^−4^–1.57 × 10^−3^), while pairwise analysis at nucleotide and amino acid levels revealed an average identity of 99.93–99.98% and 99.86–99.97%, respectively. Given the short divergence time, a high level of identity is to be expected; however, a few synonymous and non-synonymous substitutions were observed in the ORF1ab, S, OFR3a M, ORF8, N, and OFR10 genes (Figures 3A,B). When pairwise distances were calculated based on gene length, the highest levels of divergence were observed within genes ORF10 and ORF8 when considering synonymous and non-synonymous substitutions, respectively (Figures 3C,D).

Also, the estimated per site evolutionary rate in the coding regions revealed that 98.85% of the sites in the genome are evolving at expected rates of evolution, while 1.15% of the sites are evolving faster than expected (Figure 3E). In this context, and consistent with the length of the OFR1ab gene, most of these synonymous and non-synonymous substitutions (82 sites) were distributed among different protein-encoding segments of this gene; the segment encoding nsp3 had the highest number of polymorphic sites (Figure 3E).

### Detection of Purifying and Diversifying Selection

Once we identified fast-evolving positions within different genes of SARS-CoV-2, we used a combination of different algorithms centered on a codon-based phylogenetic framework to detect specific codons evolving under natural selection. Overall, no recombination events potentially affecting the results of these analyses were detected using the GARD algorithm.

**Figure 4:**
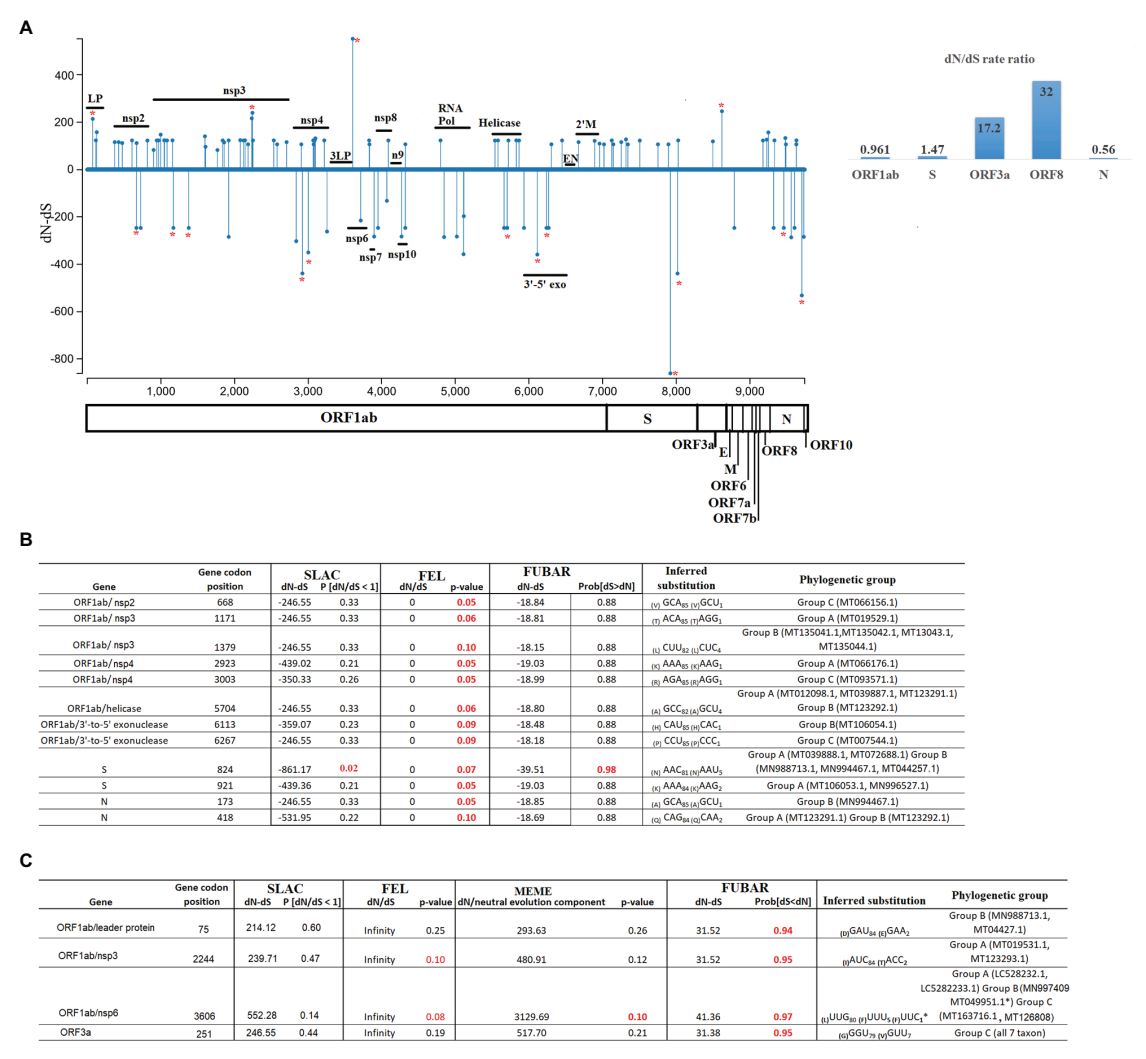
Diversifying and purifying selection on SARS-CoV-2. **(A)** General overview obtained by SLAC analysis, showing the evolutionary rate (dN-dS or dN/dS) along the genome and at individual genes of SARS-CoV-2. Statistically significant codons were inferred by multiple evolutionary tests used in this study. Red asterisks represent codons with significant evidence for selection. Codons evolving at **(B)** purifying (negative) or **(C)** diversifying (positive) selection are shown numbers in red represent evolutionary tests with significant values according to the analysis: SLAC, FEL, MEME (*p* = 0.1), and FUBAR (posterior probability = 0.9). The criteria for considering a site positively or negatively selected was based on their identification by at least one of the tests. The phylogenetic group column (assigned according with Figure 2A) shows also the isolates carrying the substitutions. LP, leader protein; 3LP, 3C-like proteinase; n9, nsp9; 3'-5' exo, 3' to 5' exonuclease; EN, endoRNAse; and 2'M, 2'-O-ribose methyltransferase.

Using SLAC, we obtained a broad picture of the extent of natural selection acting upon the SARS-CoV-2 genome. We found an overall dN/dS ratio of 0.937 along the genome. In particular, 75 codons located within five genes (ORF1ab > S > N > ORF8 > ORF3a) showed evidence of increased fixation of non-synonymous mutations (dN/dS >1). Conversely, a small number of codons (35 codons) located within five genes (ORF1ab > N > S > M = ORF10) were accumulating a higher number of synonymous mutations (dN/dS < 1). Interestingly, evaluation of dN/dS at the level of individual genes showed higher ratios for the ORF3a and ORF8 genes (Figure 4A).

Significant purifying (negative) selection was observed in 12 out of the 35 codons evolving at dN/dS < 1 using the FEL (12 sites), SLAC (1 site), and FUBAR (1 site) methods; the codons were located in the ORF1ab, S, and N genes (Figure 4B). At these codons, increased fixation of synonymous substitutions seems to be favoring the phenotypic preservation of SARS-CoV-2 at specific residues of the proteins encoded by these genes. Interestingly, negative selection of codon position 84 was the only codon supported by statistical significant values of all three methods, highlighting the relevance of this result.

Furthermore, by tracking these mutations within different isolates, we observed that these changes could explain the divergence of different viruses within different phylogenetic groups. In some cases, mutations were associated with multiple isolates, supporting the relevance of these findings.

On the other hand, evidence of diversifying positive selection on non-synonymous sites was detected in just 4 of the 75 codons evolving at dN/dS > 1 in genes ORF1ab and ORF3a, with the FUBAR and FEL methods providing the highest power of detection (Figure 4C). Based on this analysis, these four sites appear to be evolving under pervasive diversifying selection.

Interestingly, all sites detected under positive selection were found in at least two isolates, and in case of codon 3606 (nsp6), positive selection was significantly supported by three different tests. Also, the selection of this site was observed in isolates from all three phylogenetic groups, thus supporting the reliability of these findings. As a way to assess the nature of different amino acid substitutions, we used the Blosum score. In two cases at ORF1ab codons 75 (D–E = 2) and 3,606 (L–F = 1) replacements were made between amino acids with similar biological characteristics. Conversely, at codons 251 of the ORF3a (G–V; BSM62 = −3) and ORF1ab codon 2,244 (I–T; BSM62 = −1) replacements were made between amino acids of different biological proprieties. Interestingly, change at codon position 251 is highly conserved within isolates of group C, suggesting that this change might have promoted the divergence of this group.

### Detection of Directional Selection

**Figure 5:**
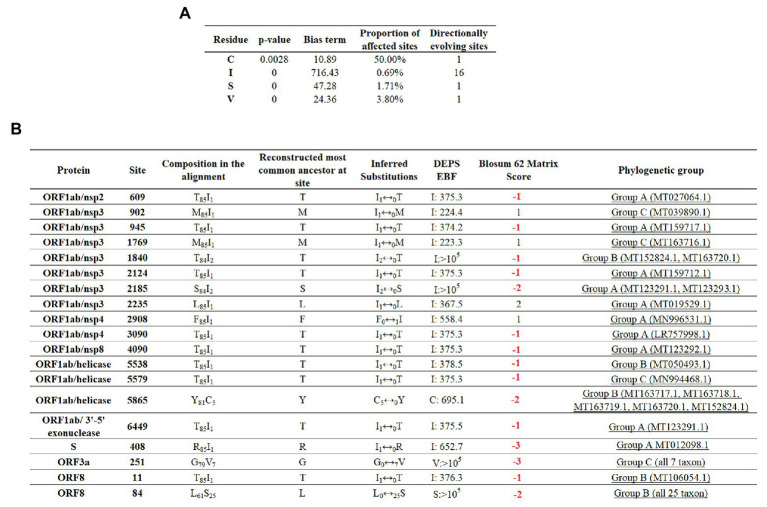
Directional selection analysis on SARS-CoV-2. **(A)** An amino acid alignment was evaluated by DEPS and four different residues producing 19 directionally evolving sites in the proteome of SARS-CoV-2 are reported. Values of *p* show the statistical significance of each residue considering a model test of selection vs. not selection. Bias term: alignment-wide relative rate of substitution toward target residue. Proportion of affected sites: percentage of sites evolving under a directional model vs. a standard model with no directionality. Directionally evolving sites: number of sites that show evidence of directional selection for focal residue. **(B)** Description of 19 directionally evolving sites. Sites were detected by Empirical Bayesian Factor (EBF) considering a cut-off of 100 or more. Numbers in red represent replacements between amino acids with different properties. The phylogenetic group column (assigned according with Figure 2A) shows also the isolates carrying the substitutions.

To maximize the inference of potential sites experiencing positive selection, amino acid alignments of SARS-CoV-2 were analyzed using the DEPS algorithm. Overall, DEPS identified a total of four amino acid residues that are experiencing directional selection. Of these four residues, isoleucine (I) has the strongest bias, affecting 16 out of 19 sites evolving *via* directional selection (Figure 5A).

The majority of selected sites were located in nonstructural proteins (nsp) encoded by the ORF1ab gene, with nsp3 accounting for the highest proportion (Figure 5B). Overall, just a low proportion of the total number of predicted sites resulted in a conservative amino acid substitution (residues at positions 902, 1,769, 2,235, and 2,908). Remarkable, among those residues experiencing replacements between amino acids with different biological properties, residue 84 of protein ORF8 appeared to be synapomorphic in all Group B sequences. Also, similar to previous algorithms, DEPS identified positive selection of residue 251 of ORF3a, supporting the potential significance of this site in the early evolution of SARS-CoV-2.

### Evidence of Coevolution Among Sites

**Figure 6:**
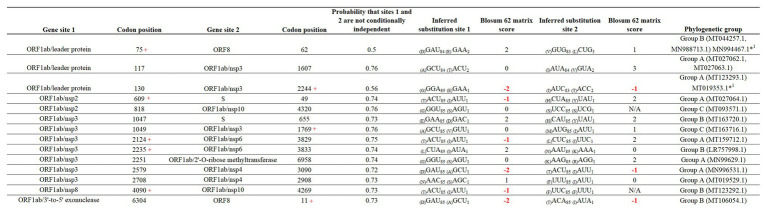
Coevolution between codon pairs in the genome of SARS-CoV-2. BMG analysis was conducted to detect coevolving codon pairs. Evidence of 14 coevolving codon pairs was detected and the specific locations of those in the genome of SARS-CoV-2 are presented. Posterior probability of pair associations was supported by Markov Chain Monte Carlo Analysis at cut-off of 50 or more. Numbers in red represent replacements between amino acids with different properties. The phylogenetic group column (assigned according with Figure 2A) shows also the isolates carrying the substitutions. *^1^Represents viral isolated where the changes were not detected. Red + represents codons under positive selection, in which coevolution with other codon might represent and epistatic event.

Finally, we attempted to find coevolutionary correlations between different codons within the genome that result in the positive selection of sites. Analysis by BMG produced evidence of 14 coevolving codon pairs; these interactions took place mostly within codons located within the ORFB1ab gene (Figure 6). Although most of the interactions were detected between nonsynonymous codons, coevolution between codons 4,090–4,269 and 818–4,320 was detected by a synonymous substitution at one of the codons. Also, based on the nature of the amino acid replacement, just 6 of the 14 interactions resulted in replacements between amino acids with different biological properties. Interestingly, 8 of the 14 interactions appeared associated with sites evolving under some type of positive selection, suggesting that the selection of these sites might be the result of epistatic events.

## Discussion

Herein, we evaluated the phylogenetic and evolutionary dynamics of SARS-CoV-2 during the first month of the pandemic event in 2020. Our phylogenetic analysis revealed the complex dynamic of the spread of infection throughout the world, suggesting that multiple viral variants might have emerged in China before the start of the pandemic event. The evolutionary rate calculated for SARS-CoV-2 in this study was consistent with previous reports for SARS-CoV (Salemi et al., 2004; Zhao et al., 2004), explaining the high levels of identity at nucleotide and amino acid levels calculated for SARS-CoV-2 in our study. In this context, the high conservation observed in the genome of SARS-CoV-2 early during the pandemic might also be attributed to the unique RNA correction machinery of coronaviruses (Ferron et al., 2018).

However, and despite the relative genome stability observed in SARS-CoV-2 at this stage of the pandemic, we were able to describe the existence of at least three phylogenetic groups. Interestingly, these findings are consistent with the results of a previous research published just 2 days before the submission of our manuscript (Forster et al., 2020). Nevertheless, both studies have significant methodological differences, which increases the reliability of the results obtained. First, different datasets were analyzed in both studies: sequences reported by Forster et al. (2020), were obtained from The Global Initiative on Sharing Avian Influenza and Coronavirus public-private partnership database (GISAID).^3^ Second, although similar cut-offs were considered for the analysis of viral sequences (spanning for December 2019 to March 4 and 10, 2020 for Forster et al., 2020 and ours, respectively), our analysis was conducted with about half the number of sequences used by Forster et al. (2020). Since both studies revealed similar clustering patterns, it may indicate that the sequences sampled in the present study accurately represent the existent diversity. Lastly, the methodologies employed used to infer the early evolutionary events of SARS-CoV-2 were different. In the present study, a Bayesian phylogenetic analysis was carried out (Nascimento et al., 2017). Forster et al. (2020) utilized a phylogenetic network analysis (Bandelt et al., 1999), an alternative methodology used to visually represent the evolutionary relationships between different taxa, when the levels of data incongruence are large (Schliep et al., 2017). Although phylogenetic tree based methods appear as the most common analytical choice, the use of one of the two methodologies can be justified based on the evolutionary complexity of the data (Schliep et al., 2017). Also, the similarity between the our results and the ones obtained by Forster et al. (2020) can help to clarify some concerns regarding the usefulness of the phylogenetic networks to infer the evolution of SARS-CoV-2 (Sanchez-Pacheco et al., 2020).

In this context, we consider that the limited number of variable sites in the genome of SARS-CoV-2 at the early phase of the pandemic might represent a real challenge for different phylogenetic reconstruction methods. As a part of our research, multiple attempts to infer early evolutionary trends in the evolution of SARS-CoV-2 were conducted using neighbor joining and maximum likelihood approaches. Still, in both cases, these methods failed to give optimal clade resolution and significant statistical support to the evolutionary inferences (data not shown). Conversely, we found that Bayesian phylogenetic analysis offers a good alternative to reconstruct the evolutionary trends of SARS-CoV-2.

Furthermore, we supported the results of our phylogenetic analysis by conducted a *F*_ST_ analysis. This analysis has been applied to infer population structure in other RNA viruses like deformed wing virus, Israel acute paralysis virus (Cornman et al., 2013), black-streaked dwarf virus (Zu et al., 2019), and rice stripe virus (Wei et al., 2009). Since the interpretation of statistically significant *F*_ST_ values can vary among different species, being, for example, values between 0.05 and 0.2 considered significant for mammal populations (vonHoldt et al., 2016), we decided to use a conservative value for viral populations (>0.33; Wei et al., 2009; Zu et al., 2019). The average *F*_ST_ values of 0.51 depicted between the pair comparisons among all three different SARS-CoV-2 phylogenetic groups indicates that 50% of the genetic variation in the SARS-CoV-2 population analyzed in our study might be attributed to genetic differentiation rather than genetic flow.

Given the presumed origin of SARS-CoV-2 (Andersen et al., 2020), where the infectious cycle in nature of these viruses is mostly maintained between bats or rats and domestic or wild animals (Ye et al., 2020), it may be expected the existence of early events of divergence in SARS-CoV-2 as a result of adaptation to human populations. In this context, similar results were seen during the evolution of the epidemic of SARS-CoV, where phylogenetic analysis shows the existence of early evolutionary events during this epidemic is possible to see that strains originated form early and middle phases of the epidemic event showed higher diversity (appearing in distinct phylogenetic clusters) than strains originated late during the epidemic, thus supporting the hypothesis that multiple strains originated the epidemic event of SARS-CoV (Yip et al., 2009).

Interestingly, our evolutionary analysis supported the hypothesis regarding that early divergence events produced during the pandemic of SARS-CoV-2 might be associated with positive selection of specific sites at ORF3a and ORF8. By using a combination of different evolutionary algorithms, we attempted to maximize the detection of codon sites that may be promoting the divergence of SARS-CoV-2 by diversifying or directional selection. In this sense, two primary considerations must be addressed regarding the biological relevance of multiple sites detected in this study. First, a considerable number of polymorphisms were detected in just one viral isolate, which might be a consequence of the small number of viral isolates available at the time we started this study. Another possibility is that some of the polymorphisms might be due to sequencing errors. Second, it is important to consider that some of the codons detected as a positive selected sites might have been product of a false positive results, since all these algorithms are not exempt of this issue (Murrell et al., 2013).

In this context, MEME might be expected to be the most sensitive test since it can detect both pervasive and episodic selection (Spielman et al., 2019). However, in our analysis, we observed a superior performance of FUBAR over the other three codon-based tests. This fact is consistent with a previous research showing that FUBAR is expected to have a better performance over SLAC and FEL over most circumstances (Murrell et al., 2013), being also methodological differences between different algorithms another factor to explain the differences observed in our study (Spielman et al., 2019). On the other hand, despite of the discrepancies among different algorithms, in all cases, positive codons detected by FUBAR showed large dN-dS values with borderline *p*-values in the other tests. Hence, we may assume that these sites are likely to be under diversifying selection. Also, as previously reported (Kosakovsky Pond et al., 2008), we observed that the addition of DEPS as a part of our methodology increased the detection of potential important residues of SARS-CoV-2 by detecting sites evolving under directional selection, suggesting that the combination of both FUBAR and DEPS may be used to support future evolutionary analysis of SARS-CoV-2.

Additionally, we used the Blosum 62 matrix score to evaluate if physicochemical properties of different amino acid replacements were preserved or modified by the evolutionary process. Interestingly, we observed larger negative scores indicating changes in the physicochemical properties on selected residues, promoting the divergence among three different phylogenetic groups (ORF3a V251G, score 3, and ORF8 L84S, score 2), indicating that these changes may produce potential effects in the function of these proteins. Conversely, positive selection of residue 3,606 [ORF1ab was associated with a conservative replacement (L–F, score 1)]. However, and despite the nature of this replacement, a previous amino acid stability analysis in SARS-CoV-2 indicated that this change might confer lower stability to the nsp6 structure, thus potentially affecting viral autophagy (Benvenuto et al., 2020). In all cases, experimental evidence is needed to define the relevance of these findings in SARS-CoV-2.

Based on the results of different evolutionary algorithms, and supported by the number of sequences affected by these polymorphisms, we are reporting four potentially relevant residues that may be driving the early evolution of SARS-CoV-2 in human populations. Firstly, the positive selection of residue 3606 (nsp6) supported by three different tests, indicating that this residue is under strong pervasive diversifying selection, affecting isolates from three different phylogenetic groups. As explained above, this change might be relevant for the virulence of SARS-CoV-2 since the function of nsp6 in different coronavirus is implicated in limiting autophagosome expansion, potentially favoring viral infection by limiting the delivery of viral proteins for degradation (Cottam et al., 2014).

Secondly, residue 251 of the ORF3a protein appears to be positive selected by FUBAR and DEPS tests, suggesting, as seen in influenza virus, that diversifying and directional selection processes are not mutually exclusive (Kosakovsky Pond et al., 2008). The selection of this site in SARS-CoV-2 seems to be relevant since it might be related to the emergence of viruses in phylogenetic Group C. The early selection of this site might have a biological relevance since the ORF3a protein has been associated with virulence of human coronaviruses by controlling not only the expression of cytokines and chemokines but also inducing necrotic cell death (Shi et al., 2019). In fact, a recent publication comparing the ability of ORF3a proteins between SARS-CoV and SARS-CoV-2 to induce apoptosis indicates that ORF3a of SARS-CoV-2 decreases levels of apoptosis in infected cells, potentially allowing the virus to spread more widely during the infection (Ren et al., 2020).

Thirdly, a residue located at position 84 of the ORF8 protein was found to be evolving under directional selection and might be related to the emergence of Group B. Mutations at ORF8 might be highly relevant since this protein has been implicated in viral pathogenesis by regulating the initial innate response in SARS-CoV (McBride and Fielding, 2012; Shi et al., 2019). In this context, a potential mechanism in which ORF8 can regulate the virulence of SARS-CoV-2 might be associated with its ability to interact MCH-1 molecules to downregulate their surface expression in different cell types, disrupting antigen presentation and viral clearance by cytotoxic cells (Park, 2020; Zhang et al., 2020). Furthermore, in terms of evolution, and based on the notably increased dN-dS values, our results indicate that early during the pandemic, the ORF8 gene was under intense evolutionary pressure. These results are consistent with SARS-CoV evolutionary signatures, suggesting that ORF8 might facilitate cornaviruses-host shifts (Forni et al., 2017). Conversely, relaxed purifying selection rather than positive selection must be considered an alternative to explaining the high dN-dS values in ORF8 observed in our study. This evolutionary signature was already described in the evolution of ORF8 gene during the epidemic of SARS-CoV, suggesting that this gene might not have had an important adaptative role during this epidemic (Forni et al., 2017). Further studies are needed to confirm these findings in SARS-CoV-2.

Additionally, we found that residue 5,865 (ORF1ab/helicase) is evolving under directional selection and might be related to the divergence of five isolates from Washington, United States, forming a sub-cluster in Group B. The relevance of this residue in SARS-CoV2 helicase’s ability to inhibit interferon production in infected cells (Yuen et al., 2020) remains to be established.

Finally, our analysis of coevolution revealed some potential epistatic interactions that might be driving the evolution of SARS-CoV-2. This mechanism has been proposed to explain the emergence of an Ebola virus variant in 2014 (Ibeh et al., 2016), and its relevance in the evolution of coronaviruses should be explored in future studies. Also, it is interesting to mention that most of the co-evolving sites were located in nsp3; given the role of this protein in the virulence of coronaviruses (Fehr et al., 2015), this observation may be key in understanding the evolution of SARS-CoV-2. Furthermore, since two of the interactions detected by BGM were associated with synonymous mutations, the relevance of this type of substitution to viral fitness should not be underestimated, since selection of synonymous substitutions has been reported in other RNA viruses like VSV (Novella et al., 2004; Velazquez-Salinas et al., 2018).

Collectively, our results describe the early evolutionary events of SARS-CoV-2 during the current pandemic and the findings may support the hypothesis that different variants of SARS-CoV-2 might be circulating in the world. However, in the absence of experimental work showing phenotypic differences among different isolates of SARS-CoV-2, we cannot rule out the alternative hypothesis claiming that early events of divergence of SARS-CoV-2 might have been the product of founder effects (Chookajorn, 2020; Mavian et al., 2020). In this context, the results reported in our research must be taken with caution.

## Data Availability Statement

The datasets presented in this study can be found free available in the NCBI database (https://www.ncbi.nlm.nih.gov/nucleotide/). For accession numbers see information in Figure 1.

## Author Contributions

LV-S and SZ conceived and designed the experiments. LV-S, SZ, and SE performed the experiments. LV-S, SZ, SE, DG, IN, and MB analyzed the data and wrote the manuscript. All authors contributed to the article and approved the submitted version.

### Conflict of Interest

The authors declare that the research was conducted in the absence of any commercial or financial relationships that could be construed as a potential conflict of interest.

## Footnotes

^1^https://www.who.int/emergencies/diseases/novel-coronavirus-2019

^2^http://tree.bio.ed.ac.uk/software/figtree

^3^https://www.gisaid.org/

## Supplementary Material

Supplementary Figure 1 Substitution model calculations for SARS-CoV-2.

Supplementary Figure 2 Overview about the evolutionary selection algorithms used in this study.

## References

[ref1] Alm E. Broberg E. K. Connor T. Hodcroft E. B. Komissarov A. B. Maurer-Stroh S.. (2020). Geographical and temporal distribution of SARS-CoV-2 clades in the WHO European region, January to June 2020. Euro Surveill. 25:2001410. 10.2807/1560-7917.ES.2020.25.32.2001410, PMID: 32794443 PMC7427299

[ref2] Andersen K. G. Rambaut A. Lipkin W. I. Holmes E. C. Garry R. F. (2020). The proximal origin of SARS-CoV-2. Nat. Med. 26, 450–452. 10.1038/s41591-020-0820-9, PMID: 32284615 PMC7095063

[ref3] Bandelt H. J. Forster P. Rohl A. (1999). Median-joining networks for inferring intraspecific phylogenies. Mol. Biol. Evol. 16, 37–48. 10.1093/oxfordjournals.molbev.a026036, PMID: 10331250

[ref4] Benvenuto D. Angeletti S. Giovanetti M. Bianchi M. Pascarella S. Cauda R.. (2020). Evolutionary analysis of SARS-CoV-2: how mutation of non-structural protein 6 (NSP6) could affect viral autophagy. J. Infect. 81, e24–e27. 10.1016/j.jinf.2020.03.058, PMID: 32283146 PMC7195303

[ref5] Chookajorn T. (2020). Evolving COVID-19 conundrum and its impact. Proc. Natl. Acad. Sci. U. S. A. 117, 12520–12521. 10.1073/pnas.2007076117, PMID: 32381737 PMC7293660

[ref6] Cornman R. S. Boncristiani H. Dainat B. Chen Y. Vanengelsdorp D. Weaver D.. (2013). Population-genomic variation within RNA viruses of the western honey bee, *Apis mellifera*, inferred from deep sequencing. BMC Genomics 14:154. 10.1186/1471-2164-14-154, PMID: 23497218 PMC3599929

[ref7] Coronaviridae Study Group of the International Committee on Taxonomy of Viruses (2020). The species severe acute respiratory syndrome-related coronavirus: classifying 2019-nCoV and naming it SARS-CoV-2. Nat. Microbiol. 5, 536–544. 10.1038/s41564-020-0695-z, PMID: 32123347 PMC7095448

[ref8] Cottam E. M. Whelband M. C. Wileman T. (2014). Coronavirus NSP6 restricts autophagosome expansion. Autophagy 10, 1426–1441. 10.4161/auto.29309, PMID: 24991833 PMC4203519

[ref9] Delport W. Poon A. F. Frost S. D. Kosakovsky Pond S. L. (2010). Datamonkey 2010: a suite of phylogenetic analysis tools for evolutionary biology. Bioinformatics 26, 2455–2457. 10.1093/bioinformatics/btq429, PMID: 20671151 PMC2944195

[ref10] Drummond A. J. Suchard M. A. Xie D. Rambaut A. (2012). Bayesian phylogenetics with BEAUti and the BEAST 1.7. Mol. Biol. Evol. 29, 1969–1973. 10.1093/molbev/mss075, PMID: 22367748 PMC3408070

[ref11] Fehr A. R. Athmer J. Channappanavar R. Phillips J. M. Meyerholz D. K. Perlman S. (2015). The nsp3 macrodomain promotes virulence in mice with coronavirus-induced encephalitis. J. Virol. 89, 1523–1536. 10.1128/JVI.02596-14, PMID: 25428866 PMC4300739

[ref12] Ferron F. Subissi L. Silveira De Morais A. T. Le N. T. T. Sevajol M. Gluais L.. (2018). Structural and molecular basis of mismatch correction and ribavirin excision from coronavirus RNA. Proc. Natl. Acad. Sci. U. S. A. 115, E162–E171. 10.1073/pnas.1718806115, PMID: 29279395 PMC5777078

[ref13] Forni D. Cagliani R. Clerici M. Sironi M. (2017). Molecular evolution of human coronavirus genomes. Trends Microbiol. 25, 35–48. 10.1016/j.tim.2016.09.001, PMID: 27743750 PMC7111218

[ref14] Forster P. Forster L. Renfrew C. Forster M. (2020). Phylogenetic network analysis of SARS-CoV-2 genomes. Proc. Natl. Acad. Sci. U. S. A. 117, 9241–9243. 10.1073/pnas.2004999117, PMID: 32269081 PMC7196762

[ref15] Frost S. D. W. Magalis B. R. Kosakovsky Pond S. L. (2018). Neutral theory and rapidly evolving viral pathogens. Mol. Biol. Evol. 35, 1348–1354. 10.1093/molbev/msy088, PMID: 29688481 PMC6279309

[ref16] Hasegawa M. Kishino H. Yano T. (1985). Dating of the human-ape splitting by a molecular clock of mitochondrial DNA. J. Mol. Evol. 22, 160–174. 10.1007/BF02101694, PMID: 3934395

[ref17] Henikoff S. Henikoff J. G. (1992). Amino acid substitution matrices from protein blocks. Proc. Natl. Acad. Sci. U. S. A. 89, 10915–10919. 10.1073/pnas.89.22.10915, PMID: 1438297 PMC50453

[ref18] Hudson R. R. Slatkin M. Maddison W. P. (1992). Estimation of levels of gene flow from DNA sequence data. Genetics 132, 583–589. PMID: 1427045 10.1093/genetics/132.2.583 PMC1205159

[ref19] Huelsenbeck J. P. Ronquist F. (2001). MRBAYES: Bayesian inference of phylogenetic trees. Bioinformatics 17, 754–755. 10.1093/bioinformatics/17.8.754, PMID: 11524383

[ref20] Ibeh N. Nshogozabahizi J. C. Aris-Brosou S. (2016). Both epistasis and diversifying selection drive the structural evolution of the Ebola virus glycoprotein mucin-like domain. J. Virol. 90, 5475–5484. 10.1128/JVI.00322-16, PMID: 27009964 PMC4934740

[ref21] Kosakovsky Pond S. L. Frost S. D. (2005). Not so different after all: a comparison of methods for detecting amino acid sites under selection. Mol. Biol. Evol. 22, 1208–1222. 10.1093/molbev/msi105, PMID: 15703242

[ref22] Kosakovsky Pond S. L. Poon A. F. Leigh Brown A. J. Frost S. D. (2008). A maximum likelihood method for detecting directional evolution in protein sequences and its application to influenza A virus. Mol. Biol. Evol. 25, 1809–1824. 10.1093/molbev/msn123, PMID: 18511426 PMC2515872

[ref23] Kosakovsky Pond S. L. Posada D. Gravenor M. B. Woelk C. H. Frost S. D. (2006). GARD: a genetic algorithm for recombination detection. Bioinformatics 22, 3096–3098. 10.1093/bioinformatics/btl474, PMID: 17110367

[ref24] Kumar S. Stecher G. Tamura K. (2016). MEGA7: molecular evolutionary genetics analysis version 7.0 for bigger datasets. Mol. Biol. Evol. 33, 1870–1874. 10.1093/molbev/msw054, PMID: 27004904 PMC8210823

[ref25] Mavian C. Pond S. K. Marini S. Magalis B. R. Vandamme A. M. Dellicour S.. (2020). Sampling bias and incorrect rooting make phylogenetic network tracing of SARS-COV-2 infections unreliable. Proc. Natl. Acad. Sci. U. S. A. 117, 12522–12523. 10.1073/pnas.2007295117, PMID: 32381734 PMC7293693

[ref26] McBride R. Fielding B. C. (2012). The role of severe acute respiratory syndrome (SARS)-coronavirus accessory proteins in virus pathogenesis. Viruses 4, 2902–2923. 10.3390/v4112902, PMID: 23202509 PMC3509677

[ref27] Murrell B. Moola S. Mabona A. Weighill T. Sheward D. Kosakovsky Pond S. L.. (2013). FUBAR: a fast, unconstrained bayesian approximation for inferring selection. Mol. Biol. Evol. 30, 1196–1205. 10.1093/molbev/mst030, PMID: 23420840 PMC3670733

[ref28] Murrell B. Wertheim J. O. Moola S. Weighill T. Scheffler K. Kosakovsky Pond S. L. (2012). Detecting individual sites subject to episodic diversifying selection. PLoS Genet. 8:e1002764. 10.1371/journal.pgen.1002764, PMID: 22807683 PMC3395634

[ref29] Nascimento F. F. Reis M. D. Yang Z. (2017). A biologist’s guide to Bayesian phylogenetic analysis. Nat. Ecol. Evol. 1, 1446–1454. 10.1038/s41559-017-0280-x, PMID: 28983516 PMC5624502

[ref30] Novella I. S. Zarate S. Metzgar D. Ebendick-Corpus B. E. (2004). Positive selection of synonymous mutations in vesicular stomatitis virus. J. Mol. Biol. 342, 1415–1421. 10.1016/j.jmb.2004.08.003, PMID: 15364570

[ref31] Park M. D. (2020). Immune evasion via SARS-CoV-2 ORF8 protein? Nat. Rev. Immunol. 20:408. 10.1038/s41577-020-0360-z, PMID: 32504060 PMC7273379

[ref32] Phan M. V. T. Ngo Tri T. Hong Anh P. Baker S. Kellam P. Cotten M. (2018). Identification and characterization of Coronaviridae genomes from Vietnamese bats and rats based on conserved protein domains. Virus Evol. 4:vey035. 10.1093/ve/vey035, PMID: 30568804 PMC6295324

[ref33] Pond S. L. Frost S. D. Muse S. V. (2005). HyPhy: hypothesis testing using phylogenies. Bioinformatics 21, 676–679. 10.1093/bioinformatics/bti079, PMID: 15509596

[ref34] Poon A. F. Lewis F. I. Pond S. L. Frost S. D. (2007). An evolutionary-network model reveals stratified interactions in the V3 loop of the HIV-1 envelope. PLoS Comput. Biol. 3:e231. 10.1371/journal.pcbi.0030231, PMID: 18039027 PMC2082504

[ref35] Rambaut A. Drummond A. J. Xie D. Baele G. Suchard M. A. (2018). Posterior summarization in Bayesian phylogenetics using tracer 1.7. Syst. Biol. 67, 901–904. 10.1093/sysbio/syy032, PMID: 29718447 PMC6101584

[ref36] Ren Y. Shu T. Wu D. Mu J. Wang C. Huang M.. (2020). The ORF3a protein of SARS-CoV-2 induces apoptosis in cells. Cell. Mol. Immunol. 17, 881–883. 10.1038/s41423-020-0485-9, PMID: 32555321 PMC7301057

[ref60] Ronquist F. Huelsenbeck J. P. (2003). MrBayes 3: Bayesian phylogenetic inference under mixed models. Bioinformatics 19, 1572–1574. 10.1093/bioinformatics/btg180, PMID: 12912839

[ref37] Salemi M. Fitch W. M. Ciccozzi M. Ruiz-Alvarez M. J. Rezza G. Lewis M. J. (2004). Severe acute respiratory syndrome coronavirus sequence characteristics and evolutionary rate estimate from maximum likelihood analysis. J. Virol. 78, 1602–1603. 10.1128/jvi.78.3.1602-1603.2004, PMID: 14722315 PMC321409

[ref38] Salmona M. Caporossi A. Simmonds P. Thelu M. A. Fusillier K. Mercier-Delarue S.. (2016). First next-generation sequencing full-genome characterization of a hepatitis C virus genotype 7 divergent subtype. Clin. Microbiol. Infect. 22, 947.e941–947.e948. 10.1016/j.cmi.2016.07.032, PMID: 27515394

[ref39] Sanchez-Pacheco S. J. Kong S. Pulido-Santacruz P. Murphy R. W. Kubatko L. (2020). Median-joining network analysis of SARS-CoV-2 genomes is neither phylogenetic nor evolutionary. Proc. Natl. Acad. Sci. U. S. A. 117, 12518–12519. 10.1073/pnas.2007062117, PMID: 32381733 PMC7293637

[ref40] Schliep K. Potts A. J. Morrison D. A. Grimm G. W. (2017). Intertwining phylogenetic trees and networks. Methods Ecol. Evol. 8, 1212–1220. 10.1111/2041-210X.12760

[ref41] Shi C. S. Nabar N. R. Huang N. N. Kehrl J. H. (2019). SARS-coronavirus open reading frame-8b triggers intracellular stress pathways and activates NLRP3 inflammasomes. Cell Death Discov. 5:101. 10.1038/s41420-019-0181-7, PMID: 31231549 PMC6549181

[ref42] Simmonds P. (2012). SSE: a nucleotide and amino acid sequence analysis platform. BMC Res. Notes 5:50. 10.1186/1756-0500-5-50, PMID: 22264264 PMC3292810

[ref43] Spielman S. J. Weaver S. Shank S. D. Magalis B. R. Li M. Kosakovsky Pond S. L. (2019). Evolution of viral genomes: interplay between selection, recombination, and other forces. Methods Mol. Biol. 1910, 427–468. 10.1007/978-1-4939-9074-0_14, PMID: 31278673

[ref44] Sun P. Qie S. Liu Z. Ren J. Li K. Xi J. (2020). Clinical characteristics of hospitalized patients with SARS-CoV-2 infection: a single arm meta-analysis. J. Med. Virol. 92, 612–617. 10.1002/jmv.25735, PMID: 32108351 PMC7228255

[ref46] Velazquez-Salinas L. Pauszek S. J. Stenfeldt C. O’hearn E. S. Pacheco J. M. Borca M. V.. (2018). Increased virulence of an epidemic strain of vesicular stomatitis virus is associated with interference of the innate response in pigs. Front. Microbiol. 9:1891. 10.3389/fmicb.2018.01891, PMID: 30158915 PMC6104175

[ref47] vonHoldt B. M. Cahill J. A. Fan Z. Gronau I. Robinson J. Pollinger J. P.. (2016). Whole-genome sequence analysis shows that two endemic species of North American wolf are admixtures of the coyote and gray wolf. Sci. Adv. 2:e1501714. 10.1126/sciadv.1501714, PMID: 29713682 PMC5919777

[ref48] Wan Y. Shang J. Graham R. Baric R. S. Li F. (2020). Receptor recognition by the novel coronavirus from Wuhan: an analysis based on decade-long structural studies of SARS coronavirus. J. Virol. 94, e00127–e00120. 10.1128/JVI.00127-20, PMID: 31996437 PMC7081895

[ref49] Wang Y. Wang Y. Chen Y. Qin Q. (2020). Unique epidemiological and clinical features of the emerging 2019 novel coronavirus pneumonia (COVID-19) implicate special control measures. J. Med. Virol. 92, 568–576. 10.1002/jmv.25748, PMID: 32134116 PMC7228347

[ref50] Weaver S. Shank S. D. Spielman S. J. Li M. Muse S. V. Kosakovsky Pond S. L. (2018). Datamonkey 2.0: a modern web application for characterizing selective and other evolutionary processes. Mol. Biol. Evol. 35, 773–777. 10.1093/molbev/msx335, PMID: 29301006 PMC5850112

[ref51] Wei T. Y. Yang J. G. Liao F. L. Gao F. L. Lu L. M. Zhang X. T.. (2009). Genetic diversity and population structure of rice stripevirus in China. J. Gen. Virol. 90, 1025–1034. 10.1099/vir.0.006858-0, PMID: 19264655

[ref52] Weiss S. R. Navas-Martin S. (2005). Coronavirus pathogenesis and the emerging pathogen severe acute respiratory syndrome coronavirus. Microbiol. Mol. Biol. Rev. 69, 635–664. 10.1128/MMBR.69.4.635-664.2005, PMID: 16339739 PMC1306801

[ref53] Wu F. Zhao S. Yu B. Chen Y. M. Wang W. Song Z. G.. (2020). A new coronavirus associated with human respiratory disease in China. Nature 579, 265–269. 10.1038/s41586-020-2008-3, PMID: 32015508 PMC7094943

[ref54] Ye Z. W. Yuan S. Yuen K. S. Fung S. Y. Chan C. P. Jin D. Y. (2020). Zoonotic origins of human coronaviruses. Int. J. Biol. Sci. 16, 1686–1697. 10.7150/ijbs.45472, PMID: 32226286 PMC7098031

[ref55] Yip C. W. Hon C. C. Shi M. Lam T. T. Chow K. Y. Zeng F.. (2009). Phylogenetic perspectives on the epidemiology and origins of SARS and SARS-like coronaviruses. Infect. Genet. Evol. 9, 1185–1196. 10.1016/j.meegid.2009.09.015, PMID: 19800030 PMC7106296

[ref56] Yuen C. -K. Lam J. -Y. Wong W. -M. Mak L. -F. Wang X. Chu H.. (2020). SARS-CoV-2 nsp13, nsp14, nsp15 and orf6 function as potent interferon antagonists. Emerg. Microbes Infect. 9, 1418–1428. 10.1080/22221751.2020.1780953, PMID: 32529952 PMC7473193

[ref57] Zhang Y. Zhang J. Chen Y. Luo B. Yuan Y. Huang F.. (2020). The ORF8 protein of SARS-CoV-2 mediates immune evasion through potently downregulating MHC-I. bioRxiv [Preprint]. 10.1101/2020.05.24.111823

[ref58] Zhao Z. Li H. Wu X. Zhong Y. Zhang K. Zhang Y. P.. (2004). Moderate mutation rate in the SARS coronavirus genome and its implications. BMC Evol. Biol. 4:21. 10.1186/1471-2148-4-21, PMID: 15222897 PMC446188

[ref59] Zu H. Zhang H. Yao M. Zhang J. Di H. Zhang L.. (2019). Molecular characteristics of segment 5, a unique fragment encoding two partially overlapping ORFs in the genome of rice black-streaked dwarf virus. PLoS One 14:e0224569. 10.1371/journal.pone.0224569, PMID: 31697693 PMC6837423

