# Supplementary figures and images for: Positive Selection of ORF1ab, ORF3a, and ORF8 Genes Drives the Early Evolutionary Trends of SARS-CoV-2 During the 2020 COVID-19 Pandemic
Source: Front Microbiol. 2020 Oct 23;11:550674. doi: 10.3389/fmicb.2020.550674 (PMC7644918; doi:10.3389/fmicb.2020.550674)

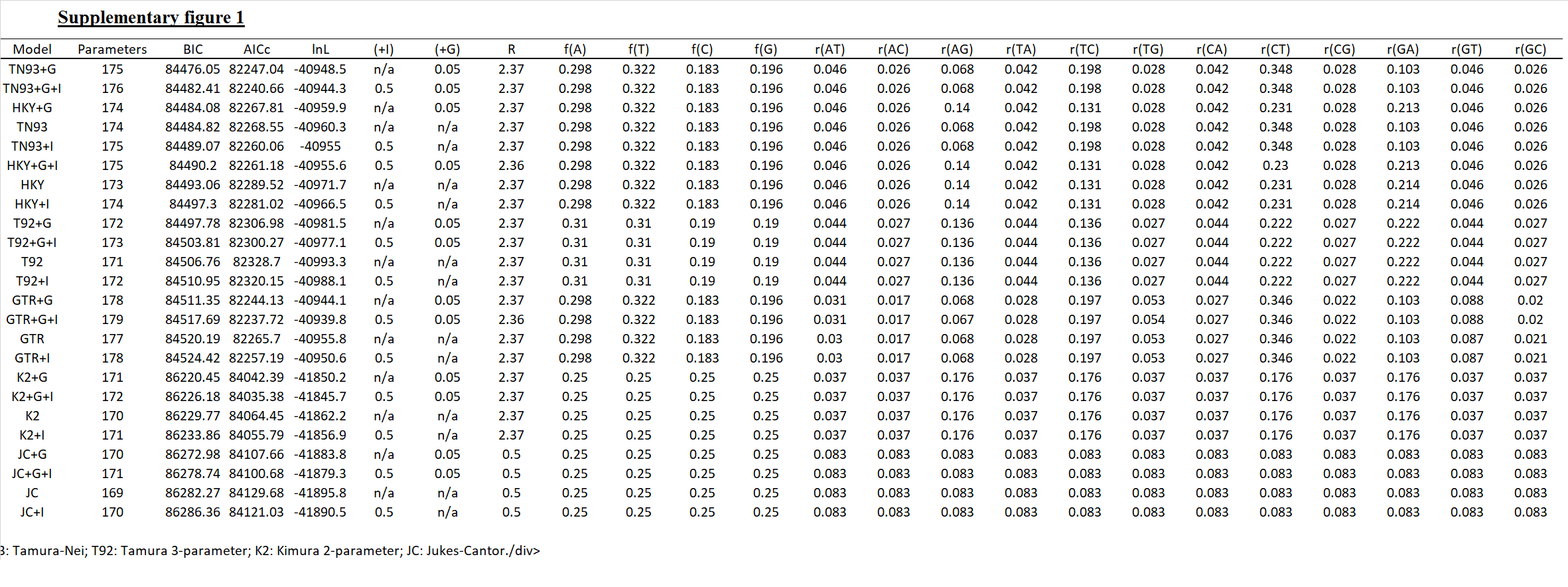

Supplement: Supplementary Figure 1 — Substitution model calculations for SARS-CoV-2. [file Image_1.tif]

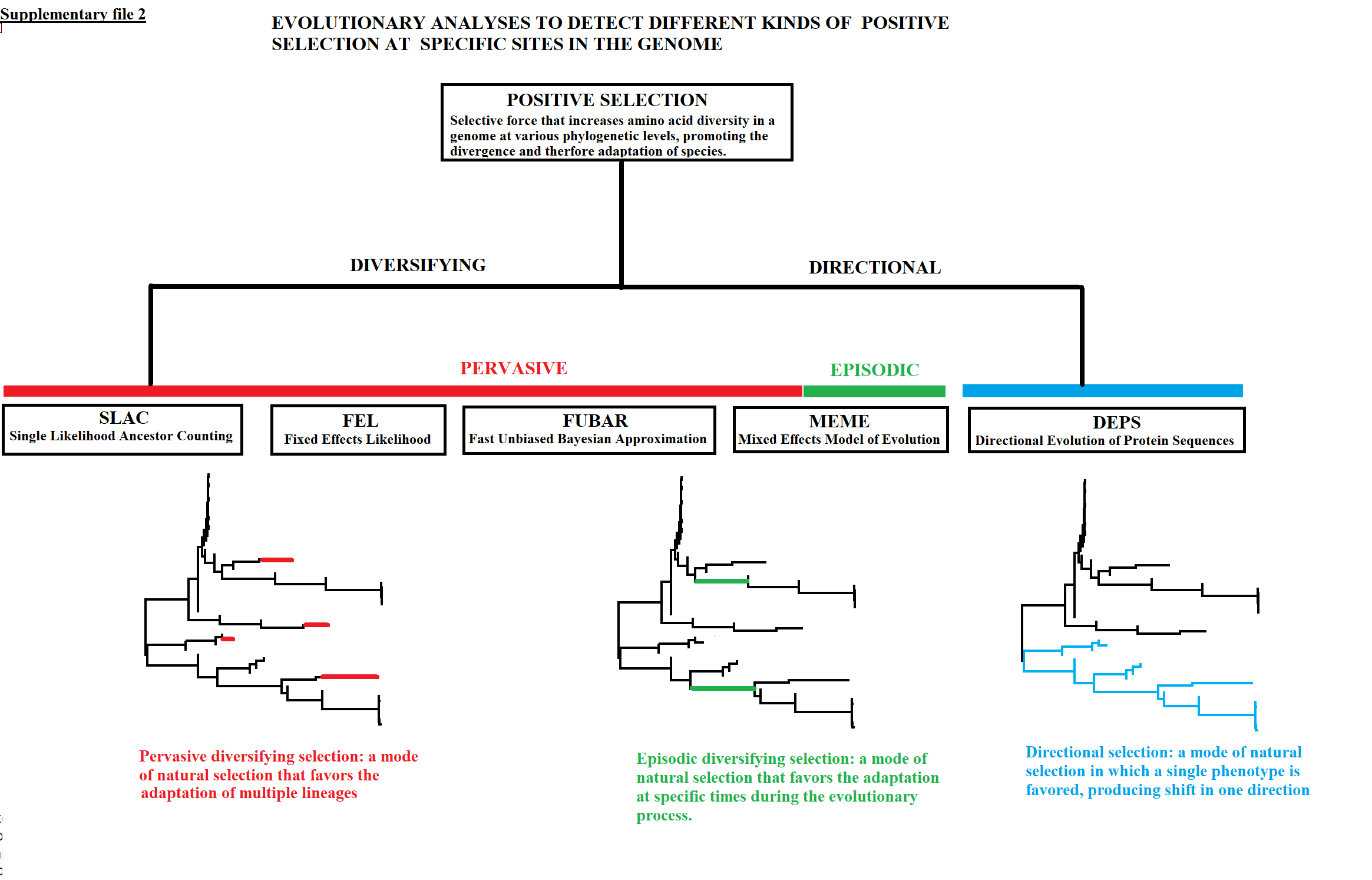

Supplement: Supplementary Figure 2 — Overview about the evolutionary selection algorithms used in this study. [file Image_2.tif]
